# Fine Mapping Reveals That Promotion Susceptibility Locus 1 (*Psl1*) Is a Compound Locus With Multiple Genes That Modify Susceptibility to Skin Tumor Development

**DOI:** 10.1534/g3.113.009688

**Published:** 2014-04-03

**Authors:** Joe M. Angel, Erika L. Abel, Penny K. Riggs, S. Alex McClellan, John DiGiovanni

**Affiliations:** * Division of Pharmacology & Toxicology, College of Pharmacy, The University of Texas at Austin, Austin, Texas 78712; † Department of Biology, Baylor University, Waco, Texas 76798; ‡ Department of Animal Science, Texas A&M University, College Station, Texas 77843; § Department of Nutritional Sciences, College of Natural Science, The University of Texas at Austin, Austin, Texas 78712

**Keywords:** Psl1, skin tumor promotion susceptibility locus, tetradecanoylphorbol acetate, complex trait genetics, compound locus

## Abstract

Although it is well known that the majority of human cancers occur as the result of exposure to environmental carcinogens, it is clear that not all individuals exposed to a specific environmental carcinogen have the same risk of developing cancer. Considerable evidence indicates that common allelic variants of low-penetrance, tumor susceptibility genes are responsible for this interindividual variation in risk. We previously reported a skin tumor promotion susceptibility locus, *Psl1*, which maps to the distal portion of chromosome 9, that modified skin tumor promotion susceptibility in the mouse. Furthermore, *Psl1* was shown to consist of at least two subloci (*i.e.*, *Psl1.1* and *Psl1.2*) and that glutathione S-transferase alpha 4 (*Gsta4*), which maps to *Psl1.2*, is a skin tumor promotion susceptibility gene. Finally, variants of human *GSTA4* were found to be associated with risk of nonmelanoma skin cancer. In the current study, a combination of nested and contiguous C57BL/6 congenic mouse strains, each inheriting a different portion of the *Psl1* locus from DBA/2, were tested for susceptibility to skin tumor promotion with 12-*O*-tetradecanoylphorbol-13-acetate. These analyses indicate that *Psl1* is a compound locus with at least six genes, including *Gsta4*, that modify skin tumor promotion susceptibility. More than 550 protein-coding genes map within the *Psl1* locus. Fine mapping of the *Psl1* locus, along with two-strain haplotype analysis, gene expression analysis, and the identification of genes with amino acid variants, has produced a list of fewer than 25 candidate skin tumor promotion susceptibility genes.

Human cancers can be divided into two major categories, familial cancers and sporadic cancers (reviewed in Angel *et al.* 2010). Familial cancers occur within affected families at a relatively high frequency and include a number of cancer syndromes that are caused by germline mutations of genes with strong effects that are directly involved in tumorigenesis. In contrast, the majority of human cancers are sporadic forms that result from exposure to environmental carcinogens (reviewed in Clavel 2007). Recent epidemiologic studies (Lichtenstein *et al.* 2000; Peto and Mack 2000; Pharoah *et al.* 2002), as well as studies that use animal models of cancer (reviewed in Angel *et al.* 2010), have shown that common allelic variants within the genome can act to modify relative risk of cancer development resulting from environmental carcinogen exposure.

Genes that modify susceptibility to tumor development (hereafter referred to as tumor susceptibility genes) are low-penetrance genes with modest effects on cancer susceptibility that are involved in DNA repair, immune response, carcinogen metabolism, cellular proliferation, differentiation, and death, as well as other cancer-related mechanisms. The combined effects of multiple tumor susceptibility genes determine the overall susceptibility of an individual to the development of a particular type of cancer, with each gene acting to either increase or decrease susceptibility. Although the identification of specific genes that underlie tumor susceptibility loci has been difficult, genes that underlie several modifier loci, including *Psl1.2* (*Gsta4*), *Mom1* (*Pla2g2a*), *Pctr1* (*Cdkn2a*), *Skts13* (*Aurka*), *Skts14* (*Tgfb1*), *Mtes1* (*Sipa1*), and others have recently been identified and variants that affect both function and expression have been reported (reviewed in Angel *et al.* 2010). Importantly, polymorphisms in these genes have also been associated with cancer risk in humans, demonstrating the utility of using animal models to identify genes that modify susceptibility to cancer (reviewed in Angel *et al.* 2010).

The multistage model of mouse skin carcinogenesis is an excellent model for the study of human epithelial carcinogenesis, and genetic differences in susceptibility to multistage skin carcinogenesis have been known for many years (Boutwell 1976; Slaga 1984; Naito and Digiovanni 1989; Digiovanni 1992). Our laboratory, as well as others, has identified genetic loci that modify susceptibility to skin tumor promotion by 12-*O*-tetradecanoylphorbol-13-acetate (TPA), showing that susceptibility to skin tumor promotion is a multigenic trait (Nagase *et al.* 1995; Angel *et al.* 1997; Mock *et al.* 1998; Angel and Digiovanni 1999; Nagase *et al.* 1999; Angel *et al.* 2001; Peissel *et al.* 2001; Angel *et al.* 2003; Fujiwara *et al.* 2007). We previously mapped TPA promotion susceptibility loci to chromosomes (chr) 1 (*Psl3*), 2 (*Psl2*), 9 (*Psl1*), and 19 (*Psl4*) in genetic crosses of C57BL/6 with DBA/2 mice (Angel *et al.* 1997; Angel and Digiovanni 1999; Angel *et al.* 2001, 2003) and recently reported that *Psl1* consists of at least two subloci, *Psl1.1* and *Psl1.2* (Abel *et al.* 2010). From these earlier studies, glutathione S-transferase alpha 4 (*Gsta4*) was identified as a skin tumor promotion susceptibility gene that maps within the *Psl1.2* locus (Abel *et al.* 2010). Furthermore, polymorphisms in *GSTA4* were found to be associated with risk of developing nonmelanoma skin cancer in humans (Abel *et al.* 2010). In the current study, analyses of combinations of nested and contiguous subcongenic mouse strains suggest that at least two additional modifier genes underlie the *Psl1.1* locus and that at least four modifier genes, including *Gsta4*, underlie the *Psl1.2* locus, indicating that *Psl1* is a compound quantitative trait locus (QTL). Additionally, inheritance of the DBA/2 allele of four of these loci (*Psl1.1a*, *Psl1.2a*, *Psl1.2b*, *Gsta4*) results in increased sensitivity whereas inheritance of the DBA/2 allele of two loci (*Psl1.1b*, *Psl1.2c*) results in decreased sensitivity to skin tumor promotion. Analysis of genes with expression or amino acid variants that map to regions of nonequivalency [chromosome regions that are not identical by descent (IBD)] within these loci has led to the identification of several attractive candidate skin tumor promotion susceptibility genes.

## Materials and Methods

### Mice

**Figure 1:**
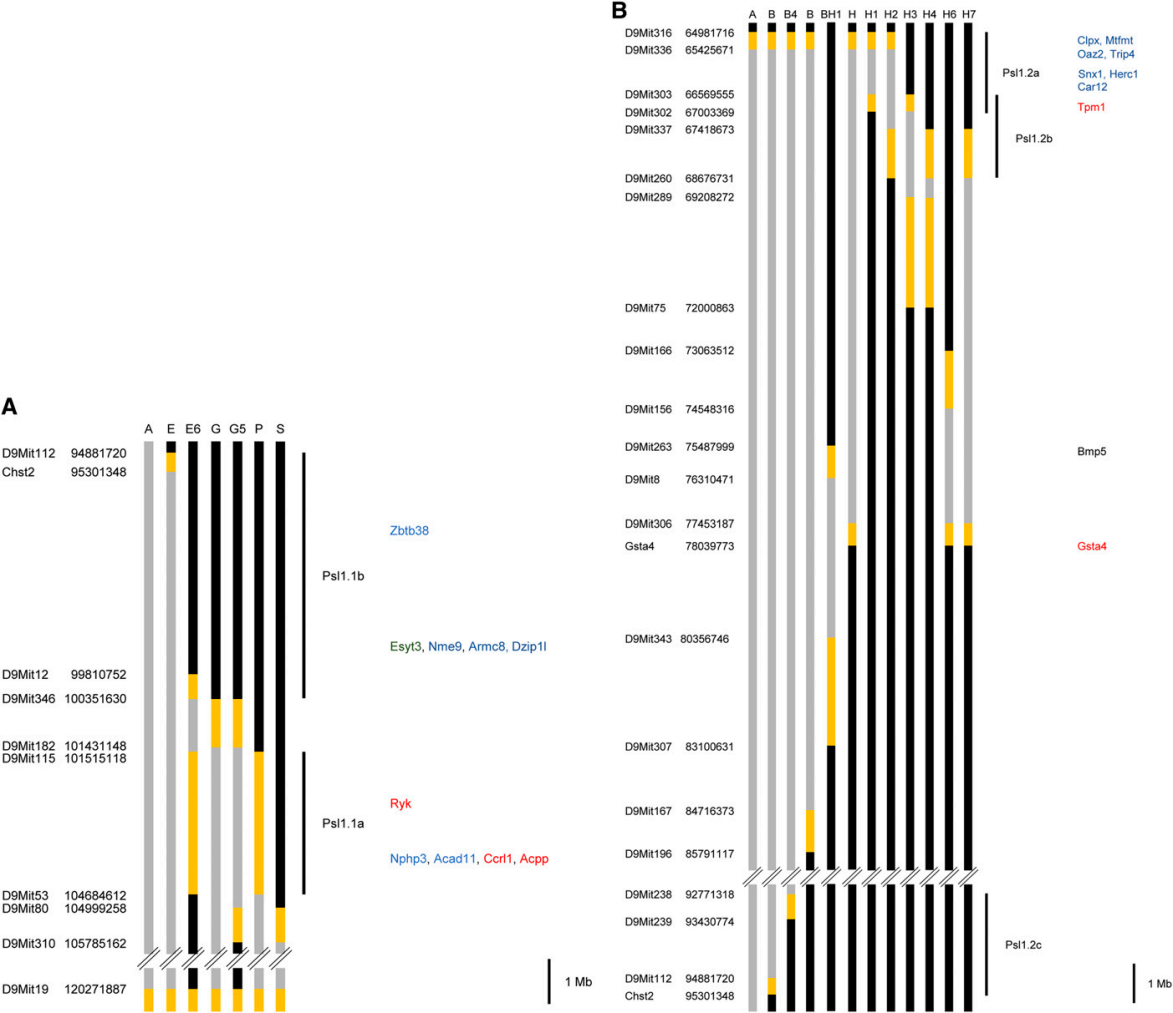
Distal chromosome 9 haplotype maps of C57BL/6.Psl1^dba^ subcongenic strains used to fine map the (A) *Psl1.1* and (B) *Psl1.2* loci. Subcongenic mouse strain designations are indicated at the top of each figure. Gray bars indicate chromosomal regions inherited from DBA/2. Black bars indicate chromosomal regions inherited from C57BL/6. Yellow bars indicate chromosomal regions with unknown genotype. Genetic markers used to genotype the congenic strains are listed on the left along with map locations in bases from the centromere. Lines to the right of the maps indicate the minimal region of interest for each *Psl1* sublocus. Gene symbols on the far right of the maps show the approximate map location of genes discussed in the paper. Those in red have expression variants, those in blue have amino acid variants, and those in green have both expression and amino acid variants.

C57BL/6 and DBA/2 mice were obtained from the Jackson Laboratory (Bar Harbor, ME). C57BL/6.Psl1^dba^ congenic mouse strains (Figure 1, Table 1, and Table 2) were developed as previously described (Abel *et al.* 2010) and maintained by brother/sister mating. All mice were maintained in a specific pathogen-free environment in the vivarium at the University of Texas M.D. Anderson Cancer Center, Science Park—Research Division in accordance with institutional guidelines. Mice were housed five per plastic cage in an air-conditioned room at 24° ± 2°, had free access to food and water, and were exposed to a 12-hr light and dark cycle.

**Table 1:** Tumor response of C57BL/6.Psl1.1 subcongenic strains

Substrain*^a^*	Introgressed Region	Length, Mb	6.8 nmol TPA			13.6 nmol TPA			
			Tumor Multiplicity*^b^*	N*^c^*	*P**^d^*	Tumor Multiplicity	N	*P**^d^*	Combined *P**^e^*
A	D9Mit316 - telomere	59.0	2.4 ± 1.6	30	0.0179	8.6 ± 3.2	29	<0.0001	<0.0001
E	Chst2 - telomere	29.2	1.8 ± 1.5	30	0.3314	4.4 ± 2.8	30	0.3686	>0.1
E6	D9Mit12 - D9Mit53	4.9	3.2 ± 2.1	30	0.0005	ND			
G	D9Mit346 - telomere	23.7	3.0 ± 2.1	30	0.0017	5.6 ± 2.7	30	0.0315	0.0005
G5	D9Mit346 - D9Mit310	5.4	2.4 ± 1.5	29	0.0054	ND			
P	D9Mit115 - telomere	22.5	4.4 ± 3.9	30	0.0003	6.5 ± 4.4	30	0.0526	0.0002
S	D9Mit80 - telomere	19.0	2.0 ± 1.6	29	0.2151	5.7 ± 3.4	30	0.1123	>0.1
C57BL/6			1.6 ± 1.2	30		4.4 ± 2.3	29		

DMBA, 7,12-dimethylbenz[a]anthracene; TPA, 12-*O*-tetradecanoylphorbol-13-acetate; ND, not done.

a Mice were initiated with 100 nmol DMBA and promoted twice weekly with 6.8 nmol TPA for 40 wks or 13.6 nmol TPA for 35 wks.

b Tumors/mouse ± SD.

c Number of mice at risk.

d Significance level of tumor multiplicity compared to C57BL/6. Not adjusted for multiple testing.

e *P* values combined using the Fischer’s combined probability test (d.f. = 4). Not adjusted for multiple testing.

**Table 2:** Tumor response of C57BL/6.Psl1.2 subcongenic strains

Substrain*^a^*	Introgressed Region	Length, Mb	Tumor Multiplicity*^b^*	N*^c^*	P*^d^*
A	D9Mit316 - telomere	59.00	2.0 ± 1.9	30	0.005
B	D9Mit316 - Chst2	30.60	0.8 ± 0.7	26	>0.1
B4	D9Mit316 - D9Mit239	28.40	1.5 ± 1.6	30	0.04
B9	D9Mit316 - D9Mit196	20.80	1.5 ± 1.6	30	0.03
BH1	D9Mit263 - D9Mit307	7.60	1.9 ± 1.6	30	0.0006
H	D9Mit316 - Gsta4	13.10	1.5 ± 1.2	30	0.0072
^1^H	D9Mit316 - D9Mit302	2.00	1.3 ± 1.1	30	0.02
H2	D9Mit316 - D9Mit260	3.70	2.3 ± 1.7	17	0.0012
H3	D9Mit303 - D9Mit75	4.60	1.7 ± 2.0	30	0.02
H4	D9Mit337 - D9Mit75	5.40	2.0 ± 1.3	30	0.0001
H6	D9Mit166 - Gsta4	5.00	1.3 ± 1.4	30	0.09
H7	D9Mit337 - Gsta4	10.60	2.1 ± 1.8	30	0.001
C57BL/6			0.8 ± 1.1	30	

DMBA, 7,12-dimethylbenz[a]anthracene; TPA, 12-*O*-tetradecanoylphorbol-13-acetate.

a Mice were initiated with 100 nmol of DMBA and promoted twice weekly with 6.8 nmol TPA for 36 wks.

b Tumors/mouse ± SD.

c Number of mice at risk.

d Significance level of tumor multiplicity compared to C57BL/6. Not adjusted for multiple testing.

### Reagents

TPA was purchased from LC Laboratories (Woburn, MA), and 7,12-dimethylbenz[a]anthracene (DMBA) was purchased from Sigma-Aldrich (St. Louis, MO).

### Tumor experiments

Tumor experiments were carried out as previously described (Abel *et al.* 2009) with female mice. A standard area on the dorsal skin extending from the base of the tail to the base of the head was shaved on each mouse. Forty-eight hours later, mice were initiated by topical treatment of 100 nmol of DMBA in 0.2 mL of acetone to the shaved area. Two weeks later, mice were treated with either 6.8 or 13.6 nmol of the tumor promoter TPA twice weekly until the tumor response reached a plateau. Mice were checked weekly for the appearance of papillomas. Tumor multiplicity was calculated as the cumulative number of papillomas observed divided by the number of mice at risk when the first tumor was observed.

### Candidate genes

The list of genes located under each QTL was downloaded from Ensembl, Build 37. A gene was considered as a candidate if the gene (1) was located within a region that differed in the haplotype of C57BL/6 and DBA/2; (2) had a nonsynonymous coding polymorphism between C57BL/6 and DBA/2; or (3) was differentially expressed in the epidermis of C57BL/6 and DBA/2.

### Two-strain haplotype analysis

Single-nucleotide polymorphism (SNP) datasets of the C57BL/6 and DBA/2 inbred strains from the Imputed Diversity Array, Build 37 were used with the Mouse Strain Comparison Tool (The Center for Genome Dynamics at The Jackson Laboratory; http://cgd.jax.org/straincomparison/) to identify genetic intervals within the *Psl1* region considered equivalent (*i.e.*, IBD) between C57BL/6J and DBA/2J, where there were at least 10 consecutive SNP calls that matched. Other regions of *Psl1* were considered to be nonequivalent and genes within the nonequivalent regions were identified. Because of the high density of the SNPs and the nonuniformity of the SNP distribution within the genome, genes located within 10 Kb of the nonequivalent regions were also included.

### Nonsynonymous coding polymorphisms

The dbSNP Build 128 available from the Mouse Genome Database (*www.informatics.jax.org/*) was used to identify any nonsynonymous coding polymorphism between C57BL/6J and DBA/2J. The potential functionality of these polymorphisms was evaluated using the Sorts Intolerant From Tolerant (SIFT) tool (http://sift.jcvi.org/) (Ng and Henikoff 2001). If the polymorphism was characterized as “damaging” or it led to a stop codon, the amino acid change was considered to be functional.

### Gene expression analysis

For analysis of gene expression, the dorsal skin of female mice (7−9 wk of age) was shaved 48 hr before treatment. Mice (three mice per group) were treated twice weekly for 2 weeks topically with 0.2 mL of acetone (for controls) or TPA (6.8 nmol) in 0.2 mL of acetone. Mice were killed by cervical dislocation at various times after the final treatment and the treated skin was excised. Total RNA was extracted from the epidermis as previously described (Riggs *et al.* 2005). Expression ratios for individual genes were examined by microarray analyses (see Supporting Information, Table S1). Expression of all genes found by microarray analyses to be differentially expressed 2-fold or greater was also analyzed by quantitative reverse transcriptase polymerase chain reaction (qRT-PCR; see Table S2) to verify differential expression. In addition, expression of several genes thought to be potential candidate genes, based on their roles in carcinogenesis but not found to be differentially expressed by microarray analysis, were measured by qRT-PCR. Microarray analyses were conducted using Illumina Mouse WG-6 v2 BeadArray (Illumina, Inc., San Diego, CA). Target preparation, hybridization, and scanning were conducted by the DNA Discovery Core of the University of Tennessee Health Science Center of Genomics and Bioinformatics (University of Tennessee, Memphis) using methods suggested by the manufacturer. qRT-PCR analysis of gene expression for *Hras1* was performed using primers and probe as previously described (Riggs *et al.* 2005). qRT-PCR analyses of all other genes used TaqMan gene expression assays (see Table S3) (Applied Biosystems, Foster City, CA). For relative quantification of expression, samples were normalized to the geometric means of the levels of Hras mRNA and 18S RNA, which were previously shown to be stable and useful as reference genes in this system (Riggs *et al.* 2005).

### Statistical analysis

Statistical analyses of differences between tumor multiplicity for each mouse strain were evaluated using the Mann–Whitney *U*-test included in the Prism 5 software package (GraphPad Software, La Jolla, CA). A one-tailed test was used for analyses between tumor multiplicities of C57BL/6 and all other strains. A two-tailed test was used for analyses between tumor multiplicities between any two congenic strains. The Fisher’s combined probability test was used to evaluate the combined results of independent experiments (Table 1). Comparisons of normalized mRNA levels were evaluated using a standard two-sided Student’s *t*-test. A *P*-value of 0.05 or less was considered statistically significant.

## Results

As noted in the *Introduction*, the skin tumor promotion susceptibility locus, *Psl1*, maps to the distal half of chr 9 (Angel *et al.* 1997, 2001, 2010; Angel and Digiovanni 1999; Abel *et al.* 2010). We recently reported that when initiated with 2.5 μmol of *N*-methyl-*N*′-nitro-*N*-nitrosoguanidine (MNNG) and promoted twice weekly with 13.6 nmol of TPA for 36 weeks, the congenic mouse strain, C57BL/6.Psl1A^dba^, which inherited a 59.1-Mb region of chr 9 (distal to *D9Mit316*) from DBA/2 (Figure 1), was significantly more sensitive to skin tumor promotion than C57BL/6 controls (Abel *et al.* 2010). Similar results were observed in the current studies when C57BL/6.Psl1A^dba^ mice were initiated with 100 nmol DMBA and promoted twice weekly with either 6.8 or 13.6 nmol of TPA (Table 1 and Table 2), further supporting the previous conclusion that the genes underlying the *Psl1* locus have an effect on tumor promotion rather than tumor initiation. Furthermore, analysis of the tumor response of C57BL/6.Psl1B^dba^ and C57BL/6.Psl1E^dba^ subcongenic strains (Figure 1) initiated with 2.5 μmol of MNNG and promoted twice weekly with 13.6 nmol of TPA suggested that *Psl1* consisted of at least two subloci, *Psl1.1* and *Psl1.2*, that modify susceptibility to skin tumor promotion (Abel *et al.* 2010). To further refine the map location of these subloci, additional interval specific C57BL/6.Psl1^dba^ subcongenic mouse strains were developed (Figure 1, Table 1, and Table 2) and tested for sensitivity to skin tumor promotion by TPA.

### Fine mapping of the *Psl1.1* sublocus

To fine map the *Psl1.1* sublocus, the response of interval specific subcongenic mouse strains (Table 1 and Figure 1A) to skin tumor promotion with TPA was examined via a standard protocol. Mice were initiated with 100 nmol of DMBA and promoted twice weekly with 6.8 or 13.6 nmol of TPA until the tumor response reached a plateau (Table 1). C57BL/6.Psl1G^dba^, C57BL/6.Psl1P^dba^, and C57BL/6.Psl1E6^dba^ were significantly more sensitive than C57BL/6 mice to skin tumor promotion by TPA whereas the tumor response of C57BL/6.Psl1S^dba^ was similar to that of C57BL/6 (Table 1). These data are consistent with other studies in which mice were initiated with MNNG (J. M. Angel and J. DiGiovanni, unpublished data). These results suggest that one or more genes mapping to a 3.2-Mb region of chr 9 between *D9Mit115* and *D9Mit53* modifies susceptibility to skin tumor promotion by TPA. This locus has been designated *Psl1.1a* (Figure 1A). The observation that C57BL/6.Psl1G5^dba^ mice were significantly more sensitive than C57BL/6 mice (Table 1) to skin tumor promotion by TPA is consistent with the mapping of a locus to this region. Inheritance of *Psl1.1a* from DBA/2 results in increased sensitivity to skin tumor promotion.

C57BL/6.Psl1E^dba^ mice, which are homozygous for the DBA/2 allele of *Psl1.1a* (Figure 1A), had a tumor response similar to that of C57BL/6 when initiated with 100 nmol of DMBA and promoted twice weekly with either 6.8 or 13.6 nmol TPA (Table 1). The observations that C57BL/6.Psl1E6 mice are sensitive whereas C57BL/6.Psl1E mice are resistant to skin tumor promotion (Table 1) suggest that one or more genes mapping to a 5.5-Mb region of chr 9 between *D9Mit112* and *D9Mit346* modify susceptibility to skin tumor promotion by TPA. This locus has been designated *Psl1.1b* (Figure 1A), and inheritance of this locus from DBA/2 results in decreased sensitivity to skin tumor promotion by TPA.

### Fine mapping of the *Psl1.2* sublocus

To fine map the *Psl1.2* sublocus, additional interval specific subcongenic strains (Table 2, Figure 1B) were analyzed for their susceptibility to skin tumor promotion with TPA. Again, mice were initiated with 100 nmol of DMBA and promoted twice weekly with 6.8 nmol of TPA until the tumor response reached a plateau (Table 2). The observations that C57BL/6.Psl1BH1^dba^ mice were more sensitive than C57BL/6 mice (Table 2) and that C57BL/6.Psl1H6^dba^ mice had a tumor response similar to C57BL/6 (Table 2) are consistent with the conclusion that *Gsta4* is a tumor promotion susceptibility gene as recently reported (Abel *et al.* 2010).

C57BL/6.Psl1H^dba^ mice were also significantly more sensitive than C57BL/6 mice to skin tumor promotion by TPA. These data are consistent with other studies in which mice were initiated with MNNG (J. M. Angel and J. DiGiovanni, unpublished data) suggesting that one or more genes mapping proximal to *Gsta4* modify susceptibility to skin tumor promotion. Additional subcongenic strains were analyzed for skin tumor promotion susceptibility to further delimit this region. Both C57BL/6.Psl1H1^dba^ and C57BL/6.Psl1H3^dba^ had similar tumor responses that were significantly greater than that of C57BL/6 (Table 2). Furthermore, C57BL/6.Psl1H2^dba^ mice were significantly more sensitive to skin tumor promotion by TPA than either C57BL/6.Psl1H1^dba^ (*P* = 0.02) or C57BL/6.Psl1H3^dba^ (*P* = 0.05) mice and had a tumor response similar to the combined response of C57BL/6.Psl1H1^dba^ and C57BL/6.Psl1H3^dba^ (data not shown). Taken together, these data suggest that at least two genes that modify susceptibility to skin tumor promotion by TPA map between *D9Mit316* and *D9Mit260*. One locus, *Psl1.2a* (Figure 1B), maps to a region of chr 9 between *D9Mit316* and *D9Mit302* whereas the second locus, *Psl1.2b* (Figure 1B), maps to a region of chr 9 between *D9Mit303* and *D9Mit260*. The observations that C57BL/6.Psl1H4^dba^ and C57BL/6.Psl1H7^dba^ mice were also significantly more sensitive than C57BL/6 mice to skin tumor promotion (Table 2) suggest that the map location of *Psl1.2b* can be narrowed to a region of chr 9 between *D9Mit337* and *D9Mit260* (Figure 1B). However, the possibility that the sensitivity of these two strains is due to a third skin tumor promotion susceptibility locus mapping between *D9Mit260* and *D9Mit75* cannot be ruled out at the present time. Inheritance of either *Psl1.2a* or *Psl1.2b* from DBA/2 results in increased susceptibility to skin tumor promotion by TPA.

The tumor response of C57BL/6.Psl1B^dba^ mice was similar to that of C57BL/6 mice when initiated with 100 nmol of DMBA and promoted twice weekly with 6.8 nmol of TPA (Table 2). The observation that C57BL/6.Psl1B^dba^ mice were relatively resistant to skin tumor promotion by TPA, even though they inherited the susceptibility alleles of *Psl1.2a*, *Psl1.2b*, and *Gsta4* from DBA/2, suggests that a fourth gene mapping distal to *D9Mit306* causes a decrease in skin tumor promotion susceptibility when inherited from DBA/2. To further narrow the map location of this gene, the tumor response of two additional subcongenic mouse strains, C57BL/6.Psl1B4^dba^ and C57BL/6.Psl1B9^dba^ (Table 2, Figure 1B), was examined. Both strains were significantly more sensitive than C57BL/6 mice to skin tumor promotion by TPA (Table 2), suggesting that a resistance locus maps to a region of chr 9 between *D9Mit238* and *Chst2*. This locus has been designated *Psl1.2c* (Figure 1B).

### Characterization of the *Psl1.1a* locus

Potential candidate genes can be identified with haplotype mapping, which takes advantage of the fact that laboratory strains of mice were derived from the same founders and share regions of the genome that are IBD (Wiltshire *et al.* 2003). Haplotype blocks in which the two parental strains are not IBD are the most probable location for causal genetic variation (Wade *et al.* 2002). Two-strain haplotype analysis, comparing C57BL/6 and DBA/2 haplotypes as described in the *Materials and Methods* section, were conducted to identify regions of the *Psl1* locus that are not IBD (nonequivalent), and genes mapping to these regions were identified. Of 24 protein-coding genes that map to *Psl1.1a*, 15 map to regions of nonequivalency. In addition, one microRNA gene (*Mir2136*) and one long intergenic noncoding RNA (*Gm17708*) map to the *Psl1.1a* locus, both in regions of nonequivalency.

Different alleles of a candidate gene that underlie a QTL must display either an expression or functional variant. Twenty-two of the 24 protein-coding genes mapping to the *Psl1.1a* locus were analyzed by microarray and/or qRT-PCR for mRNA levels in the epidermis of TPA-treated C57BL/6 and DBA/2 mice 6 hr after the last of twice-weekly treatments for 2 wk. Nine of these protein-coding genes that map to regions of nonequivalency were found to be expressed above background (see Table S1 and Table S2), and three of these genes, chemokine (c-c motif) receptor-like 1 (*Ccrl1*), prostate acid phosphatase (*Accp*), and receptor-like tyrosine kinase (*Ryk*), were differentially expressed in the epidermis of C57BL/6 compared with DBA/2 mice 6 hr after the final TPA treatment (Figure 1A and Table 3). Four other genes examined by qRT-PCR were found to be differentially expressed at other time points, with mRNA levels being greater in the epidermis of C57BL/6 compared with DBA/2 mice for angiomotin-like 2 (*Amotl2*) and kyphoscoliosis peptidase (*Ky*) and greater in TPA-treated DBA/2 compared with C57BL/6 epidermis for transmembrane protein 108 (*Tmem108*) and Eph receptor B1 (*Ephb1*) (data not shown). These genes may also be candidate promotion susceptibility genes. Interestingly, *Rab6b*, which maps within a region of IBD, was also differentially expressed in the epidermis of TPA-treated C57BL/6 compared with DBA/2 mice (data not shown), suggesting that expression of this gene may be regulated in *trans*.

**Table 3:** Potential candidate genes differentially expressed in the epidermis of TPA-treated C57BL/6 *vs.* DBA/2 mice

Locus	Map Location	Gene Symbol	C57BL/DBA*^a^*	
			Microarray	qRT-PCR
Psl1.1a	102737250	Ryk	1.0	2.4
Psl1.1a	104000468	Ccrl1	1.4	5.5
Psl1.1a	104190570	Acpp	3.5	5.2
Psl1.1b	99210386	Esyt3	2.8	4.6
Psl1.2a or b	66870400	Tpm1	2.0	6.4

qRT-PCR, quantitative reverse transcription polymerase chain reaction.

a Mice were treated twice weekly for 2 wk with 6.8 nmol of TPA in 0.2 mL of acetone and killed 6 hr after the final treatment.

Comparison of DNA sequences between C57BL/6 and DBA/2 identified nonsynonymous SNPs for three protein-coding genes and one unclassified noncoding RNA gene, *Gm5627*, mapping to the *Psl1.1a* locus. Two of these genes, nephronophthisis 3 (adolescent) (*Nphp3*, Figure 1A) and acyl-Coenzyme A dehydrogenase family, member 11 (*Acad11*, Figure 1A), which were found to be expressed in the epidermis by qRT-PCR (see Table S2), were considered to be potential candidate genes. The *Nphp3* SNP was predicted to be tolerated in the SIFT database (Table 4).

**Table 4:** Potential candidate genes with nonsynonymous SNPs that map to Psl1 subloci

Locus	Map Location	Gene Symbol	SIFT
Psl1.1a	103904874	Nphp3	T
Psl1.1a	103966033	Acad11	NF
Psl1.1b	96585842	Zbtb38	T
Psl1.1b	99210386	Esyt3	NF
Psl1.1b	99356662	Nme9	T
Psl1.1b	99378810	Armc8	Damaging
Psl1.1b	99530014	Dzip1l	NF
Psl1.2a	65142102	Clpx	NF
Psl1.2a	65283589	Mtfmt	T
Psl1.2a	65515808	Oaz2	NF
Psl1.2a	65676733	Trip4	T
Psl1.2a	65935934	Snx1	NF
Psl1.2a	66198257	Herc1	T
Psl1.2a	66561493	Car12	T

SNP, single-nucleotide polymorphism; SIFT, Sorts Intolerant From Tolerant; T, tolerated; NF, not found in database.

### Characterization of the *Psl1.1b* locus

Of 49 protein-coding genes that map to *Psl1.1b*, 35 map to regions of nonequivalency. Thirty-nine of the 49 protein-coding genes mapping to the *Psl1.1b* locus were analyzed by microarray and/or qRT-PCR for mRNA levels in the epidermis of TPA-treated C57BL/6 and DBA/2 mice 6 hr after the last of twice weekly treatments for 2 wk. Twenty-one genes that map to regions of nonequivalency were found to be expressed above background (see Table S1 and Table S2). mRNA levels of one protein-coding gene, extended synaptotagmin-like protein 3 (*Esyt3*, Figure 1A), were found to be >2-fold greater in the epidermis of C57BL/6 than DBA/2 mice (Table 3). Therefore, *Esyt3* was considered to be a potential candidate gene.

Comparison of C57BL/6 and DBA/2 sequences identified six protein-coding genes with nonsynonymous SNPs mapping to the *Psl1.1b* locus. One of these genes was not expressed above background when examined by microarray. The remaining five genes (Table 4 and Figure 1A) were considered to be potential candidate genes. SNPs for three genes were found in the SIFT database (Table 4) and the nonsynonoymous SNP for one of these genes, armadillo repeat containing 8 (*Armc8*, Table 4), was predicted to be damaging.

### Characterization of the *Psl1.2a* locus

Of 36 protein-coding genes that map to *Psl1.2a*, 25 map to regions of nonequivalency. Four unclassified noncoding RNA genes map to the *Psl1.2a* locus, all in regions of nonequivalency. Thirty-three of the 36 protein-coding genes mapping to the *Psl1.2a* locus were analyzed by microarray and/or qRT-PCR for mRNA levels in the epidermis of TPA-treated C57BL/6 and DBA/2 mice 6 hr after the last of twice weekly treatments for 2 wk. Of those genes that mapped to regions of nonequivalency, 18 were expressed above background (see Table S1 and Table S2). mRNA levels of one protein-coding gene, tropomyosin 1, alpha (*Tpm1*, Figure 1B), were found to be >2-fold greater in the epidermis of C57BL/6 than DBA/2 mice (Table 3). Therefore, *Tpm1* was considered to be a potential candidate gene.

Comparison of the DNA sequences of C57BL/6 and DBA/2 identified eight protein-coding genes with nonsynonymous SNPs that map to the *Psl1.2a* locus. One of these genes was determined by microarray analysis to not be expressed above background. The remaining genes (Table 4 and Figure 1B) were considered to be candidate genes. Nonsynonymous SNPs found in four of these genes were found in the SIFT database and all four were predicted to be tolerated (Table 4).

### Characterization of the *Psl1.2b* locus

Of 12 protein-coding genes that map to *Psl1.2b*, seven map to regions of nonequivalency. One microRNA and two noncoding RNA genes map to the *Psl1.2b* locus and are found in regions of nonequivalency. Nine of the 12 protein-coding genes mapping to the *Psl1.2b* locus were analyzed by microarray for mRNA levels in the epidermis of TPA-treated C57BL/6 and DBA/2 mice 6 hr after the last of twice weekly treatments for 2 wk. Of those genes, four mapped to regions of nonequivalency and were expressed above background (see Table S1 and Table S2). mRNA levels of *Tpm1* were found to be ≥2-fold greater in the epidermis of C57BL/6 than DBA/2 mice (Table 3). *Tpm1* maps to a region of overlap for the *Psl1.2a* and *Psl1.2b* loci (Figure 1B) and was considered a potential candidate gene for either locus.

Comparison of the DNA sequences of C57BL/6 and DBA/2 identified two protein-coding genes with nonsynonymous SNPs that map to the *Psl1.2b* locus. Microarray analysis indicated that neither of these genes was expressed above background in the epidermis of acetone- or TPA-treated C57BL/6 or DBA/2 mice 6 hr after the last of four treatments.

### Characterization of the *Psl1.2c* locus

Three protein-coding genes map to the *Psl1.2c* locus, and two of these genes map to a region of nonequivalency. Neither of these genes was differentially expressed in the epidermis after the last of four treatments with TPA (see Table S1), and no nonsynonymous SNPs have been reported for either gene.

## Discussion

The skin tumor promotion susceptibility locus, *Psl1*, was previously mapped to a 59.1-Mb region of mouse chr 9 (Angel *et al.* 1997, 2001, 2010; Angel and Digiovanni 1999) and the glutathione S-transferase gene, *Gsta4*, was shown to underlie at least some of the effect of this locus on skin tumor promotion susceptibility in mice (Abel *et al.* 2010). In addition, *GSTA4* has been shown to be a modifier of susceptibility to nonmelanoma skin cancer in humans (Abel *et al.* 2010). As described in the current study, analyses of skin tumor promotion susceptibility using interval specific subcongenic mouse strains indicate that *Psl1* is a compound locus made up of a cluster of subloci (see Figure 1), similar to other QTL affecting complex traits such as blood pressure (reviewed in Rapp and Joe 2012), diabetes (Granhall *et al.* 2006), body composition (Diament and Warden 2004; Farber and Medrano 2007; Ishikawa *et al.* 2007; Prevorsek *et al.* 2010), and cancer (Samuelson *et al.* 2005, 2007). Inheritance of *Psl1.1a*, *Psl1.2a*, *Psl1.2b*, and *Gsta4* from DBA/2 results in increased susceptibility to skin tumor promotion whereas inheritance of *Psl1.1b* and *Psl1.2c* from DBA/2 results in decreased susceptibility. Although the skin tumor susceptibility locus, *Skts6*, was mapped to the distal half of chr 9 using crosses of Mus spretus with NIH/Ola mice (Nagase *et al.* 1999), it is not possible to determine from the current studies whether any of the *Psl1* subloci and *Skts6* are the same.

Of the more than 550 protein-coding genes that map within the *Psl1* locus, fewer than 25 genes that fulfill the criteria for candidate skin tumor promotion susceptibility genes were identified in the current study (summarized in Table 3 and Table 4). For example, the receptor-like tyrosine kinase, *Ryk*, maps to the *Psl1.1a* locus and is expressed 2.6-fold greater in the epidermis of TPA-treated C57BL/6 compared with DBA/2 mice (Table 3). The receptor encoded by *Ryk* functions as a co-receptor with Frizzled for Wnt ligands and binds to Dishevelled, through which it activates the canonical Wnt Pathway (Lu *et al.* 2004). A genetic variant of *RYK* has been associated with risk of breast cancer in humans (Wang *et al.* 2010), supporting the hypothesis that *Ryk* may be a candidate tumor promotion susceptibility gene.

*Ccrl1* (*CCX-CKR*) also maps to the *Psl1.1a* locus and is expressed at greater levels in TPA-treated epidermis of C57BL/6 compared with DBA/2 mice (Table 3). CCRL1 is an atypical chemokine receptor that lacks a signaling domain. Overexpression of *CCRL1* inhibited proliferation and migration of human breast cancer cells both *in vitro* and *in vivo* (Feng *et al.* 2009), supporting the hypothesis that *Ccrl1* is a cancer susceptibility gene. The CCRL1 protein selectively binds the homeostatic CC-chemokines CCL19, CCL21, and CCL25 (Gosling *et al.* 2000; Townson and Nibbs 2002; Comerford *et al.* 2010). Binding to CCRL1 results in internalization and degradation of these ligands (Comerford *et al.* 2006), which attenuates the immune response (Heinzel *et al.* 2007). Recent reports have shown that the CCRL1 ligands promote proliferation and prevent apoptosis (Wang *et al.* 2005; Li *et al.* 2009; Shen *et al.* 2009; Johnson *et al.* 2010; Xu *et al.* 2011, 2012), suggesting that increased expression of *Ccrl1* may reduce tumor response through chemokine depletion. This idea is further supported by the observation of a significantly higher rate of tumor growth in wild-type BALB/c mice compared to plt mice, which lack CCL19 and CCL21, implanted with a syngeneic squamous cell carcinoma cell line (Mburu *et al.* 2006).

Another potential candidate tumor promotion susceptibility gene, *Zbtb38* (zinc finger and BTB domain containing 38), maps to a region of nonequivalency within the *Psl1.1b* locus and has an amino acid variant between C57BL/6 and DBA/2 (Table 4). The protein encoded by *Zbtb38* is a member of the Kaiso-like family of methyl-CpG-binding proteins. It is a BTB-zinc finger transcriptional repressor that interacts with the transcriptional co-repressor C-terminal binding proteins (Sasai *et al.* 2005). C-terminal binding protein is thought to be involved in development and carcinogenesis (reviewed in Chinnadurai 2002, 2009). The protein encoded by *Zbtb38* has been identified as a substrate of caspase-3, and down-regulation induces apoptosis in mouse C2C12 cells (Oikawa *et al.* 2008). Overexpression of *Zbtb38* in mouse embryonic stem cells promotes proliferation and accelerates G1/S transition whereas small interfering RNA knockdown of expression inhibits proliferation (Nishii *et al.* 2012). Taken together, these observations suggest that *Zbtb38* may play a role in carcinogenesis. Recently, a SNP in intron 4 of *ZBTB38* was associated with prostate cancer risk in a multistage genome-wide association study (Kote-Jarai *et al.* 2011), further supporting this hypothesis.

Armadillo repeat containing 8 (*Armc8*), which also maps to a region of nonequivalency within the *Psl1.1b* locus, has an amino acid variant between C57BL/6 and DBA/2 that may be damaging to protein function (Table 4). *Armc8* encodes two splice variants that contain armadillo-repeat motifs. Armadillo-repeat containing proteins are involved in diverse cellular functions such as intracellular signaling and cell-cell contact regulation (Coates 2003). Although Armc8 has not been shown to be directly involved in tumor development, it has been shown to regulate proteasome-dependent degradation of the tumor suppressor, α-catenin (Suzuki *et al.* 2008). α-Catenin has recently been reported to regulate keratinocyte stem cell proliferation (Schlegelmilch *et al.* 2011). Loss of α-catenin triggers severe epidermal hyperplasia (Vasioukhin *et al.* 2001) and xenografts of keratinocytes lacking α-catenin produced skin lesions in nude mice that resembled squamous cell carcinomas (Kobielak and Fuchs 2006). These observations suggest that *Armc8* may modify skin tumor promotion susceptibility through altered regulation of α-catenin degradation.

Sorting nexin 1 (*Snx1*) is a potential candidate promotion susceptibility gene for the *Psl1.2a* locus (Table 4). *SNX1* is a putative tumor suppressor gene (Nguyen *et al.* 2006), and the encoded protein is a member of a large family of endocytic proteins that help determine the fate of internalized receptors as the receptors reach the early endosome (Worby and Dixon 2002). It plays a role in targeting ligand-activated epidermal growth factor receptor (EGFR) to the lysosomes for degradation after endocytosis from the cell surface and release from the Golgi (Kurten *et al.* 1996). Small interfering RNA knockdown of *SNX1* levels in tumor cell lines resulted in increased proliferation, decreased apoptosis, decreased anoikis, increased EGFR phosphorylation after EGF stimulation, and increased downstream signaling (Nguyen *et al.* 2006; Nishimura *et al.* 2012). These results suggest that *Snx1*/*SNX1* may be involved in carcinogenesis by regulating ligand-induced EGFR phosphorylation and suggest a critical function for *Snx1*/SNX1 in the maintenance of tightly regulated EGFR-mediated signaling. The C57BL/6 allele of *Snx1* encodes a protein with a serine at amino acid 117, whereas the DBA/2 allele encodes a protein with a proline at this position. This nonconserved amino acid variant maps within the phospholipid-binding-motif termed the phox homology (PX) domain, which is involved in localizing the SNX1 protein to early endosomes (Worby and Dixon 2002). A human variant of SNX1 with a point mutation (K214A) in the PX domain was incabable of binding 3-phosphoinositides and localized to the cytosol rather than to early endosomes (Cozier *et al.* 2002). Furthermore, the K214A variant could not stimulate EGFR degradation whereas the wild-type SNX1 did, indicating that the PX domain-dependent/early endosomal association of SNX1 is important for its ability to regulate the targeting of internalized EGFR for lysosomal degradation (Cozier *et al.* 2002). Additional studies will be required to determine whether the amino acid variant at position 117 in the mouse SNX1 protein has a similar effect and if this variant plays a role in skin tumor promotion susceptibility.

A recent study found that loss of expression of bone morphogenetic protein 5 (*Bmp5*), which maps to distal chr 9, was associated with reduction in size and number of clonogenic keratinocyte stem cells and an increase in sensitivity to skin tumor promotion (Kangsamaksin and Morris 2011) suggesting that *Bmp5* may underlie a portion of the effect of *Psl1* on skin tumor promotion susceptibility. However, *Bmp5* does not map to any of the *Psl1* subloci identified in the present study (Figure 1B). Furthermore, the congenic mouse strain C57BL/6.Psl1H6^dba^, which has a tumor response similar to C57BL/6 when initiated with 100 nmol of DMBA and promoted twice weekly with 6.8 nmol of TPA (Table 2), inherited the DBA/2 allele of *Bmp5* (Figure 1B), indicating that *Bmp5* does not affect skin tumor promotion susceptibility in genetic crosses of C57BL/6 with DBA/2 mice.

In conclusion, at least six genes mapping to the *Psl1* locus on distal chr 9 modify susceptibility to skin tumor promotion by TPA. Previous studies have shown that one gene that resides in this region (*Gsta4*) is a modifier of skin tumor promotion susceptibility (Abel *et al.* 2010). Other genes mapping to regions of nonequivalency within the *Psl1* subloci are either differentially expressed in the epidermis of resistant C57BL/6 *vs.* sensitive DBA/2 mice treated with TPA or have amino acid variants between the two strains of mice. Several of these genes have been associated with cancer risk in humans or play a role in signaling pathways associated with carcinogenesis, making them excellent candidate skin tumor promotion susceptibility genes. Additional studies will be required to confirm that these genes are modifiers of skin tumor promotion susceptibility.

## Supplementary Material

Supporting Information
