# Supplementary material for: Fine Mapping Reveals That Promotion Susceptibility Locus 1 (Psl1) Is a Compound Locus With Multiple Genes That Modify Susceptibility to Skin Tumor Development
Source: G3 (Bethesda). 2014 Apr 3;4(6):1071–9. doi: 10.1534/g3.113.009688 (PMC4065250; doi:10.1534/g3.113.009688)
Supplement: Supporting Information [file supp_4_6_1071__index.html]

Fine Mapping Reveals That Promotion Susceptibility Locus 1 (Psl1) Is a Compound Locus With Multiple Genes That Modify Susceptibility to Skin Tumor Development — Supporting Information 

# Fine Mapping Reveals That Promotion Susceptibility Locus 1 (*Psl1*) Is a Compound Locus With Multiple Genes That Modify Susceptibility to Skin Tumor Development

## Supporting Information for Angel *et al.*, 2014

**Files in this Data Supplement:**

- Supporting Information - Tables S1-S3 (PDF, 147 KB)
- Table S2 - Data from qRT-PCR analyses of expression of potential candidate skin tumor promotion susceptibility genes. (PDF, 112 KB)
- Table S3 - TaqMan Gene Expression Assays Used for qRT-PCR Analyses of Gene Expression. (PDF, 113 KB)
- Table S1 - Data from microarray analyses of genes mapping to regions of non-equivalency within *Psl1* subloci. (.xlsx, 25 KB)
