# Supplementary material for: Fine Mapping Reveals That Promotion Susceptibility Locus 1 (Psl1) Is a Compound Locus With Multiple Genes That Modify Susceptibility to Skin Tumor Development
Source: G3 (Bethesda). 2014 Apr 3;4(6):1071–9. doi: 10.1534/g3.113.009688 (PMC4065250; doi:10.1534/g3.113.009688)
Supplement: Supporting Information [file supp_g3.113.009688_009688SI.pdf]

G3: Genes | Genomes | Genetics

Fine mapping reveals that promotion susceptibility locus 1 (*Psl1*) is a compound locus with multiple genes that modify susceptibility to skin tumor development

Joe M. Angel[*], Erika L. Abel[§], Penny K. Riggs[†], S. Alex McClellan[*], and John DiGiovanni[*‡]

[*]Division of Pharmacology & Toxicology, College of Pharmacy, The University of Texas at Austin, Austin, Texas, 78712.

[§]Department of Biology, Baylor University, Waco, Texas, 76798.

[†]Department of Animal Science, Texas A&M University, College Station, Texas, 77843.

[‡]Department of Nutritional Sciences, College of Natural Science, The University of Texas at Austin, Austin, Texas, 78712.

Corresponding Authors:
Joe M. Angel
Dell Pediatric Research Institute
The University of Texas at Austin
1400 Barbara Jordan Blvd.
Austin, TX 78723
Fax: 512-495-4945
E-Mail: jmangel@austin.utexas.edu

John DiGiovanni
Dell Pediatric Research Institute
The University of Texas at Austin
1400 Barbara Jordan Blvd.
Austin, TX 78723
Fax: 512-495-4945
E-mail: John.digiovanni@austin.utexas.edu

DOI: 10.1534/g3.113.009688

Table S1   Data from microarray analyses of genes mapping to regions of non-equivalency within *Psl1* subloci

Available for download as an Excel file at http://www.g3journal.org/lookup/suppl/doi:10.1534/g3.113.009688/-/DC1

Table S2 Data from qRT-PCR analyses of expression of potential candidate skin tumor promotion susceptibility genes[a]

			C57BL6				DBA/2				C57BL/DBA[e]	
			Acetone		TPA		Acetone		TPA			
Gene Symbol	Psl1 Sublocus	Map Location[b]	Average dC_T[c]	SD[d]	Average dC_T	SD	Average dC_T	SD	Average dC_T	SD	Acetone Treated	TPA Treated
Tpm1	1.2a or 1.2b	66870397	29.78	1.10	32.34	0.60	32.63	0.30	35.02	0.50	7.2	6.4
Rora	1.2b	68501593	22.40	0.50	23.25	0.41	22.34	0.12	24.15	0.42	1.0	1.9
Pik3cb	1.1b	98938821	24.90	0.56	26.19	0.56	24.90	0.15	26.89	0.35	1.0	1.6
Esyt3	1.1b	99210386	30.10	0.13	29.23	0.89	30.38	0.19	31.44	0.56	1.2	4.6
Ephb1	1.1a	101824458	30.42	0.47	30.18	0.23	31.75	0.96	30.19	1.24	2.5	1.0
Ky	1.1a	102408468	28.51	0.56	32.02	1.05	27.79	0.25	32.39	0.28	0.6	1.3
Anapc13	1.1a	102528626	25.74	0.78	26.39	0.21	25.62	0.17	26.95	0.44	0.9	1.5
Amotl2	1.1a	102619002	24.37	0.46	24.75	0.26	24.39	0.17	25.28	0.23	1.0	1.4
Ryk	1.1a	102737247	26.15	0.81	26.41	0.31	26.41	0.19	27.67	0.52	1.2	2.4
Tmem108	1.1a	103385266	33.61	1.31	33.02	0.37	33.58	0.15	32.47	1.22	1.0	0.7
Nphp3	1.1a	103904874	28.33	0.37	30.01	0.47	28.02	0.12	30.46	0.26	0.8	1.4
Acad11	1.1a	103942992	25.00	0.46	26.38	0.35	25.16	0.17	26.58	0.36	1.1	1.1
Ccrl1	1.1a	104000468	30.44	0.25	33.80	0.65	31.96	0.22	36.27	0.31	2.9	5.5
Dnajc13	1.1a	104053927	28.93	0.48	28.71	0.14	28.81	0.20	29.08	0.48	0.9	1.3
Acpp	1.1a	104190581	22.84	0.56	20.41	0.34	22.75	0.07	22.81	0.82	0.9	5.2

[a]Mice (3/group) were treated twice weekly for 2 weeks with 6.8 nmol TPA and euthanized 6 h after the final treatment

[b]Bases from the centromere

[c]Values were normalized to the geometric means of reference genes *Hras* and 18s rRNA, previously shown to be stable in this system

[d]Standard Deviation

[e]Relative quantities by the method of Livak and Schmittgen (1)

1. Livak KJ, Schmittgen TD. Analysis of relative gene expression data using real-time quantitative PCR and the 2(-Delta Delta C(T)) Method. Methods. 2001;25(4):402-8. doi: 10.1006/meth.2001.1262. PubMed PMID: 11846609.

Table S3 TaqMan® Gene Expression Assays Used for qRT-PCR Analyses of Gene Expression

Gene Symbol	Gene Name	Assay
18S	18S RNA	Hs99999901_s1
Acad11	acyl-Coenzyme A dehydrogenase family, member 11	Mm00614545_m1
Acpp	acid phosphatase, prostate	Mm00480093_m1
Amotl2	angiomotin-like 2	Mm00502287_m1
Anapc13	anaphase promoting complex subunit 13	Mm01701504_g1
Ccrl1	chemokine (C-C motif) receptor-like 1	Mm02620636_s1
Dnajc13	DnaJ (Hsp40) homolog, subfamily C, member 13	Mm01224743_m1
Ephb1	Eph receptor B1	Mm00557961_m1
Esyt3	Extended synaptotagmin-like protein 3	Mm00625223_m1
Ky	kyphoscoliosis peptidase	Mm00600373_m1
Nphp3	nephronophthisis 3 (adolescent)	Mm00511917_m1
Pik3cb	phosphatidylinositol 3-kinase, catalytic, beta polypeptide	Mm00659576_m1
Rab6b	RAB6B, member RAS oncogene family	Mm00620651_m1
Rora	RAR-related orphan receptor alpha	Mm00443103_m1
Ryk	receptor-like tyrosine kinase	Mm01238551_m1
Tmem108	transmembrane protein 108	Mm00618208_m1
Tpm1	tropomyosin 1, alpha	Mm00s445895_g1
