# Supplementary material for: Fine Mapping Reveals That Promotion Susceptibility Locus 1 (Psl1) Is a Compound Locus With Multiple Genes That Modify Susceptibility to Skin Tumor Development
Source: G3 (Bethesda). 2014 Apr 3;4(6):1071–9. doi: 10.1534/g3.113.009688 (PMC4065250; doi:10.1534/g3.113.009688)
Supplement: Supporting Information [file supp_g3.113.009688_TableS2.pdf]

Table S2 Data from qRT-PCR analyses of expression of potential candidate skin tumor promotion susceptibility genes[a]

			C57BL6				DBA/2				C57BL/DBA[e]	
			Acetone		TPA		Acetone		TPA			
Gene Symbol	Psl1 Sublocus	Map Location[b]	Average dC_T[c]	SD[d]	Average dC_T	SD	Average dC_T	SD	Average dC_T	SD	Acetone Treated	TPA Treated
Tpm1	1.2a or 1.2b	66870397	29.78	1.10	32.34	0.60	32.63	0.30	35.02	0.50	7.2	6.4
Rora	1.2b	68501593	22.40	0.50	23.25	0.41	22.34	0.12	24.15	0.42	1.0	1.9
Pik3cb	1.1b	98938821	24.90	0.56	26.19	0.56	24.90	0.15	26.89	0.35	1.0	1.6
Esyt3	1.1b	99210386	30.10	0.13	29.23	0.89	30.38	0.19	31.44	0.56	1.2	4.6
Ephb1	1.1a	101824458	30.42	0.47	30.18	0.23	31.75	0.96	30.19	1.24	2.5	1.0
Ky	1.1a	102408468	28.51	0.56	32.02	1.05	27.79	0.25	32.39	0.28	0.6	1.3
Anapc13	1.1a	102528626	25.74	0.78	26.39	0.21	25.62	0.17	26.95	0.44	0.9	1.5
Amotl2	1.1a	102619002	24.37	0.46	24.75	0.26	24.39	0.17	25.28	0.23	1.0	1.4
Ryk	1.1a	102737247	26.15	0.81	26.41	0.31	26.41	0.19	27.67	0.52	1.2	2.4
Tmem108	1.1a	103385266	33.61	1.31	33.02	0.37	33.58	0.15	32.47	1.22	1.0	0.7
Nphp3	1.1a	103904874	28.33	0.37	30.01	0.47	28.02	0.12	30.46	0.26	0.8	1.4
Acad11	1.1a	103942992	25.00	0.46	26.38	0.35	25.16	0.17	26.58	0.36	1.1	1.1
Ccrl1	1.1a	104000468	30.44	0.25	33.80	0.65	31.96	0.22	36.27	0.31	2.9	5.5
Dnajc13	1.1a	104053927	28.93	0.48	28.71	0.14	28.81	0.20	29.08	0.48	0.9	1.3
Acpp	1.1a	104190581	22.84	0.56	20.41	0.34	22.75	0.07	22.81	0.82	0.9	5.2

[a]Mice (3/group) were treated twice weekly for 2 weeks with 6.8 nmol TPA and euthanized 6 h after the final treatment

[b]Bases from the centromere

[c]Values were normalized to the geometric means of reference genes *Hras* and 18s rRNA, previously shown to be stable in this system

[d]Standard Deviation

[e]Relative quantities by the method of Livak and Schmittgen (1)

1. Livak KJ, Schmittgen TD. Analysis of relative gene expression data using real-time quantitative PCR and the 2(-Delta Delta C(T)) Method. Methods. 2001;25(4):402-8. doi: 10.1006/meth.2001.1262. PubMed PMID: 11846609.
