# Supplementary material for: Fine Mapping Reveals That Promotion Susceptibility Locus 1 (Psl1) Is a Compound Locus With Multiple Genes That Modify Susceptibility to Skin Tumor Development
Source: G3 (Bethesda). 2014 Apr 3;4(6):1071–9. doi: 10.1534/g3.113.009688 (PMC4065250; doi:10.1534/g3.113.009688)
Supplement: Supporting Information [file supp_g3.113.009688_TableS3.pdf]

Table S3 TaqMan® Gene Expression Assays Used for qRT-PCR Analyses of Gene Expression

Gene Symbol	Gene Name	Assay
18S	18S RNA	Hs99999901_s1
Acad11	acyl-Coenzyme A dehydrogenase family, member 11	Mm00614545_m1
Acpp	acid phosphatase, prostate	Mm00480093_m1
Amotl2	angiomotin-like 2	Mm00502287_m1
Anapc13	anaphase promoting complex subunit 13	Mm01701504_g1
Ccrl1	chemokine (C-C motif) receptor-like 1	Mm02620636_s1
Dnajc13	DnaJ (Hsp40) homolog, subfamily C, member 13	Mm01224743_m1
Ephb1	Eph receptor B1	Mm00557961_m1
Esyt3	Extended synaptotagmin-like protein 3	Mm00625223_m1
Ky	kyphoscoliosis peptidase	Mm00600373_m1
Nphp3	nephronophthisis 3 (adolescent)	Mm00511917_m1
Pik3cb	phosphatidylinositol 3-kinase, catalytic, beta polypeptide	Mm00659576_m1
Rab6b	RAB6B, member RAS oncogene family	Mm00620651_m1
Rora	RAR-related orphan receptor alpha	Mm00443103_m1
Ryk	receptor-like tyrosine kinase	Mm01238551_m1
Tmem108	transmembrane protein 108	Mm00618208_m1
Tpm1	tropomyosin 1, alpha	Mm00s445895_g1
